# Estimation of Vehicle Dynamic Parameters Based on the Two-Stage Estimation Method

**DOI:** 10.3390/s21113711

**Published:** 2021-05-26

**Authors:** Wenfei Li, Huiyun Li, Kun Xu, Zhejun Huang, Ke Li, Haiping Du

**Affiliations:** 1 Shenzhen Institutes of Advanced Technology, Chinese Academy of Sciences, Shenzhen 518055, China; hy.li@siat.ac.cn (H.L.); kun.xu@siat.ac.cn (K.X.); zj.huang@siat.ac.cn (Z.H.); ke.li1@siat.ac.cn (K.L.); 2 CAS Key Laboratory of Human-Machine Intelligence-Synergy Systems, Shenzhen Institutes of Advanced Technology, Shenzhen 518055, China; 3 Guangdong-Hong Kong-Macao Joint Laboratory of Human-Machine Intelligence-Synergy Systems, Shenzhen 518055, China; 4 School of Electrical, Computer and Telecommunications Engineering, University of Wollongong, Wollongong 2522, Australia; hdu@uow.edu.au

**Keywords:** vehicle dynamic parameters, Unscented Kalman Filter, multiple-model

## Abstract

Vehicle dynamic parameters are of vital importance to establish feasible vehicle models which are used to provide active controls and automated driving control. However, most vehicle dynamics parameters are difficult to obtain directly. In this paper, a new method, which requires only conventional sensors, is proposed to estimate vehicle dynamic parameters. The influence of vehicle dynamic parameters on vehicle dynamics often involves coupling. To solve the problem of coupling, a two-stage estimation method, consisting of multiple-models and the Unscented Kalman Filter, is proposed in this paper. During the first stage, the longitudinal vehicle dynamics model is used. Through vehicle acceleration/deceleration, this model can be used to estimate the distance between the vehicle centroid and vehicle front, the height of vehicle centroid and tire longitudinal stiffness. The estimated parameter can be used in the second stage. During the second stage, a single-track with roll dynamics vehicle model is adopted. By making vehicle continuous steering, this vehicle model can be used to estimate tire cornering stiffness, the vehicle moment of inertia around the yaw axis and the moment of inertia around the longitudinal axis. The simulation results show that the proposed method is effective and vehicle dynamic parameters can be well estimated.

## 1. Introduction

**Figure 1:**
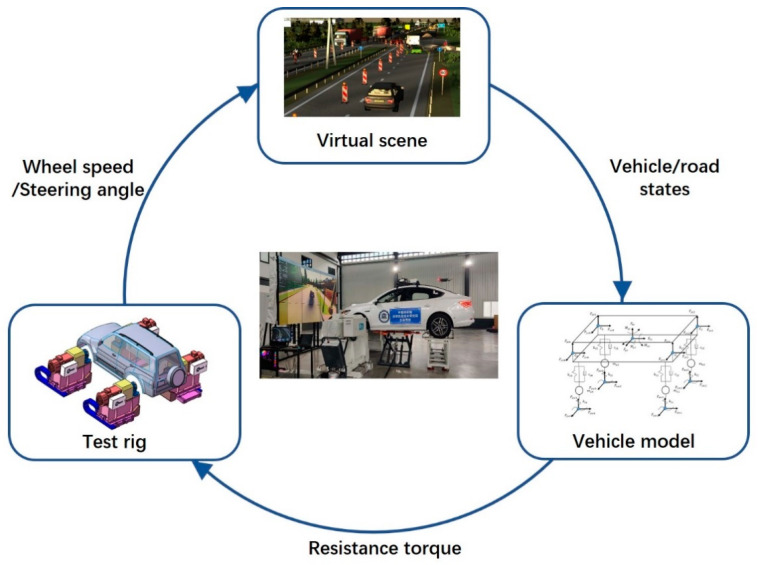
Autonomous driving test rig.

Nowadays, modern road vehicles are using an increasing number of active systems to improve vehicle safety, passenger comfort, vehicle performance and energy efficiency. Advanced Driver Assistance Systems (ADAS), as well as Automated Driving (AD) technologies, are being increasingly implemented in vehicles, aiming for improved driving safety and passenger comfort [1,2]. In addition, the autonomous driving test rig is also an important method to test autonomous driving control algorithms (as shown in Figure 1, it is an autonomous driving test rig proposed by our research group) [3,4,5]. The implementation of these fields greatly depends on accurate vehicle dynamic parameters. Vehicle dynamic parameters are also important for vehicle modeling. Thus, vehicle dynamic parameters are important for vehicle design and testing.

The vehicle dynamic parameters (VDPs), such as the vehicle mass, moment of inertia and position of the vehicle centroid, affect the closed-loop behavior of active safety systems and play an important role [6]. It is necessary to determine the VDPs to obtain real vehicle responses. Some of the VDPs can be easily measured such as the mass, the track width or the wheelbase. However, other parameters are unknown and difficult to be measured directly, such as the distance from vehicle centroid to the front axis. The moment of inertia around each axis can be measured by special equipment which is extremely costly. In contrast, the estimation method is a less intrusive and expensive way to obtain VDPs. The VDPs can be estimated by combining the estimation algorithm with some cheap sensors such as Inertial Measurement Unit (IMU), Global Positioning System (GPS), wheel speed sensors and steering angle sensor [7]. 

To obtain VDPs, many different methods have been proposed. In [8], a novel model-based parameter identification approach using optimized excitation trajectory is proposed to identify the VDPs. However, this method needs test rigs, which is a huge cost. In addition, a variety of algorithms for VDPs estimation have been presented in works of literature [9,10,11,12,13,14,15,16,17,18,19,20,21,22,23,24,25,26,27,28,29,30,31,32,33,34,35,36,37,38,39]. The influence of VDPs on vehicle dynamics often involves coupling. Most of the papers only study the estimation part of some parameters and the other parameters are treated as being easily measured or obtained. In actual applications, this strategy is not feasible. In real applications, all VDPs need to be obtained through simple sensors and estimation strategies. Since the VDPs are always coupled with the vehicle states, the state-parameter joint and dual estimation methods [9,10] have become increasingly prevalent and have been studied by many researchers. Some researchers use the Dual Kalman Filter (DKF) to identify the VDPs and the vehicle states simultaneously. Besides, VDPs estimation is usually classified based on the parameters of interest and the vehicle dynamics model used. In [11], common onboard sensors which are able to measure the lateral acceleration and yaw rate and a non-linear vehicle model are used. Augmented Extended Kalman Filtering is used to estimate motion states and tire cornering stiffness based on a non-linear vehicle model and sensor. Sideslip and roll angles of electric are estimated using lateral tire force sensors through RLS and the Kalman Filter based on the Single-track model in [12]. Sprung mass, yaw moment of inertia and longitudinal position of the center of gravity are identified through a dual unscented Kalman Filter in [13]. In [14], a four-wheel nonlinear vehicle model with roll dynamics and a correlation between the inertial parameters is used for a dual Unscented Kalman Filter to simultaneously identify the inertial parameters and the vehicle state. A local observability analysis on the nonlinear vehicle model is used to activate and deactivate different modes of the proposed algorithm. A Dual Extended Kalman Filter (DEKF) is used to estimate both vehicle states and vehicle parameters such as the vehicle mass, moment of inertia about the vertical axis and distance between the center of gravity and the front axle [15]. An extended Kalman Filter-based estimator adopting a dynamic vehicle model for determining the vehicle’s longitudinal and lateral velocity as well as the yaw rate is proposed in [16]. In [17], a novel approach based on combined *H*_∞_ and extended Kalman Filter (*H*_∞_-EKF) is used to estimate the center of gravity position of electric vehicles. To implement this estimation algorithm, a simplified vehicle dynamics model is applied to the filter formulation. The *H*_∞_ estimator is employed to filter states by means of minimizing the influence of unexpected noise, whose statistics are unknown. Simultaneously, the other EKF estimator uses the states derived by the former filter to identify the position of the vehicle centroid. A methodology based on multiple-models and a switching method for real-time estimation of the position of vehicle centroid is proposed in [13]. The method uses the well-known simple linear vehicle models for lateral and roll dynamics and assumes the availability of lateral acceleration, the yaw rate, velocity, and steering angle measurements. As mentioned in previous research, the existing estimation methods are either expensive or only portions of the VDPs can be estimated. However, vehicle dynamics modeling needs to completely determine the completed VDPs, while the cost of VDPs acquisition should be as small as possible. Thus, a method that can obtain completed VDPs at low cost urgently needs to be proposed.

In order to obtain completed VDPs at a low cost, we propose a two-stage estimation method consisting of multiple-models and Unscented Kalman Filter to estimate VDPs. In the first stage, the vehicle is set to accelerate/decelerate and the longitudinal vehicle model is used. During this stage, the height of the vehicle centroid, tire longitudinal stiffness and the longitudinal position of the vehicle centroid are estimated by the Unscented Kalman Filter. After these parameters are estimated, these estimated parameters can be used in the second stage. In the second stage, a Single-track with roll dynamics vehicle model is adopted and the vehicle is set to continuous steering. Through vehicle steering, this model can be used to estimate tire cornering stiffness, the vehicle moment of inertia around the yaw axis and the moment of inertia around the longitudinal axis. After the two-stage estimation, all VDPs are estimated. The rest of the paper is organized as follows: vehicle dynamics model are shown in Section 2. The method used to estimate VDPs is provided in Section 3. Section 4 shows and discusses the simulation results. Finally, Section 5 delivers the conclusions and points towards future work.

## 2. Vehicle Model

**Figure 2:**
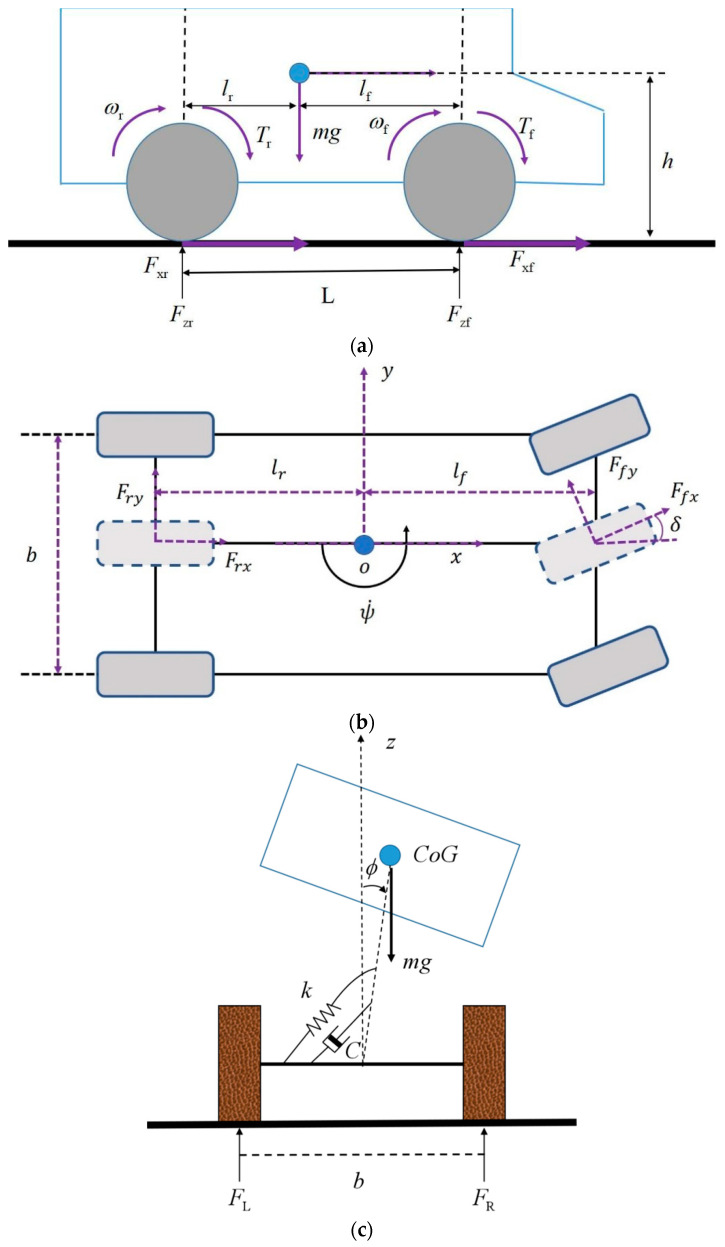
Vehicle model: (**a**) Longitudinal vehicle dynamics model; (**b**) Single-track vehicle model; (**c**) Vehicle roll dynamics.

The vehicle model used in this paper is a multiple-model approach which is based on a longitudinal vehicle dynamics model (as shown in Figure 2a) and a single-track with roll dynamics vehicle model (as shown in Figure 2b,c), which comprises: the motion in the longitudinal direction *x*, the longitudinal velocity; the motion in the lateral direction *y* or lateral velocity; the yaw around the vertical axis *z*, described by the yaw rate and roll with regard to the longitudinal axis *x*; and the roll rate [13]. Figure 2 illustrates the vehicle model adopted in this paper. The whole motion of the vehicle is a direct result of the forces (the aerodynamic forces and rolling resistance are neglected in this paper) that are generated between the road and tires. As shown in Figure 2b, the four-wheel vehicle dynamics model can be simplified as a single-track model. Other states that depend directly on these states can be derived, such as longitudinal and lateral accelerations. The tire states, such as the wheel slip angle, slip ratio and rotational velocities are also important. Tire-road friction force can be obtained based on tire states and tire stiffness. The vehicle states are also largely dependent on VDPs. VDPs include vehicle mass, moments of inertia around each axis and the position of the vehicle centroid.

The vehicle dynamic model can be described by differential equations. The vehicle model implemented here can be obtained from [17,18]. When the vehicle was accelerating or decelerating along the longitudinal direction, the longitudinal vehicle dynamics model was adopted. As shown in Figure 2a, the longitudinal vehicle dynamics model was built with the longitudinal motion, as well as the front and rear wheel rotations

(1) $$m{\dot{v}}_{x}=F_{xf}+F_{xr}$$

(2a) $$J{\dot{\omega }}_{f}=T_{f}-rF_{xf}$$

(2b) $$J{\dot{\omega }}_{r}=T_{r}-rF_{xr}$$

where m is vehicle total mass, $v_{x}$ represents the longitudinal vehicle velocity $F_{xf}$ and $F_{xr}$ represent the longitudinal forces of the front and rear tires. J is the wheel’s moment of inertia. r is the equivalent radius of the front and rear tires. $T_{i},\;\omega_{i}\;\left(i=f,r\right)$ represent the wheel torque and angular speed. The load distribution can be expressed by the vertical forces that act on each of the four wheels. These can be calculated as follows:

(3a) $$F_{zf}=mg\frac{l_{r}}{L}-ma_{x}\frac{h}{L}$$

(3b) $$F_{zr}=mg\frac{l_{f}}{L}+ma_{x}\frac{h}{L}$$

where $F_{zf}\;\mathrm{and}\;F_{zr}$ are vertical force of the front and rear wheels. $a_{x}$ is the longitudinal accelerations, g is the gravitational constant, $l_{f}$ is the distance between the vehicle centroid and vehicle front axis, $l_{r}$ is the distance between the vehicle centroid and vehicle rear axis and h denotes the height of the vehicle centroid. L is the distance between the front axis and rear axis.

When the vehicle was being steered, the single-track with roll dynamics vehicle model was adopted. As shown in Figure 1c and Figure 2b, the differential equations for the calculation of longitudinal and lateral acceleration are as follows:

(4) $${\dot{v}}_{x}=a_{x}+v_{y}\dot{\psi }$$

(5) $${\dot{v}}_{y}=a_{y}+v_{x}\dot{\psi }$$

(6) $$a_{x}=\frac{1}{m}\left(F_{xf}cos\delta +F_{yf}sin\delta +F_{xr}\right)$$

(7) $$a_{y}=\frac{1}{m}\left(F_{xf}sin\delta +F_{yf}cos\delta +F_{yr}\right)$$

Yaw and roll motion can be obtained from:

(8) $$\ddot{\psi }=\frac{\Gamma }{I_{z}}$$

(9) $$I_{x}\ddot{\phi }=mh\left(a_{y}+g\phi \right)-k_{\phi }\phi -c_{\phi }\dot{\phi }$$

where $\dot{\psi }$ is the yaw rate, $\dot{\phi }$ is the roll rate, $I_{z}$ is the moment of inertia around the yaw axis, $I_{x}$ is the moment of inertia around the longitudinal axis, $k_{\phi }$ is the roll stiffness, $c_{\phi }$ is the roll damping and $a_{y}$ is lateral acceleration. Γ can be calculated as follows:

(10) $$\Gamma =l_{f}\left(F_{xf}cos\delta +F_{yf}sin\delta \right)-l_{r}F_{yr}$$

where δ is the wheel steer angle while $F_{yr}$ represents the lateral forces of the rear tires. There are many different approaches for achieving tire force, such as the so-called ‘Magic Formula’ by Pacejka [19], the tire model by Fiala [20] or the ‘TMeasy’ tyre model [21]. When the acceleration/deceleration strength of the vehicle is small and the steering angle is small, the tire force can be calculated as follows:

(11a) $$F_{xi}=C_{\sigma }s_{i}$$

(11b) $$F_{yi}=C_{\alpha }\alpha_{i}$$

where $F_{xi},\;F_{yi}\;\left(i=f,r\right)$ represent the longitudinal and lateral tire forces, $C_{\alpha }$ denotes the tire cornering stiffness, $C_{\sigma }$ denotes the tire longitudinal stiffness, $s_{i}$ is the slip ratio and $\alpha_{i}$ is the slip angle. $\alpha_{i}$ can be presented as follows:

(12a) $$\alpha_{f}=\frac{v_{y}-l_{f}\dot{\psi }}{v_{x}}-\delta$$

(12b) $$\alpha_{r}=\frac{v_{y}-l_{r}\dot{\psi }}{v_{x}}$$

the slip ratio $s_{i}\;\left(i=f,r\right)$ can be presented as follows:

(13) $$s_{i}=\frac{\omega_{i}r}{v_{x}}-1$$

When the acceleration/deceleration strength of the vehicle was small, the tire-road friction coefficient was proportional to the slip ratio rate [21]. Then the longitudinal tire force can also be presented as follows:

(14) $$F_{xi}=F_{zi}C_{K}s_{i}$$

where $C_{K}$ is the slip ratio rate. It is a constant value related to the road surface. When the road surface was different, $C_{K}$ changed as well. From Equations (11a) and (14), it can be seen that tire longitudinal stiffness can be calculated based on the slip ratio rate and vertical force of the wheel. This means that the tire longitudinal stiffness can be obtained when the slip ratio rate is estimated.

## 3. Estimation Method

**Figure 3:**
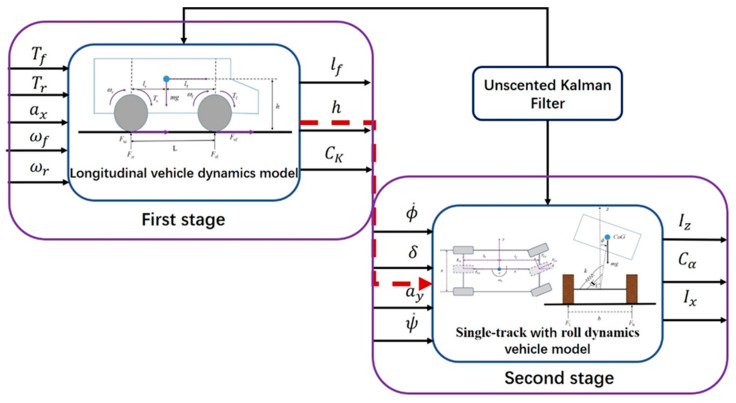
Two-stage estimation method.

To adapt to non-linear problems in vehicle dynamics estimation, EKF is widely used for estimating different vehicle states. However, the accuracy of EKF-based estimation cannot be guaranteed due to linearization errors with Jacobian matrices when approximating non-linear systems [20,21,22,23,24,25,26]. More recently, additional attention has been paid to UKF estimation, which uses a set of sigma points to conduct non-linear transformation so that it can deal with strong non-linear estimation problems for vehicle dynamics systems [31]. The UKF, developed by Julier et al. [32] and refined by Wan and van der Merwe et al. [33] provides a new estimation approach. Unlike the EKF, the UKF approximates the probability density function of system states by implementing the Unscented Transformation (UT) instead of the system dynamics model. The UT captures the mean and covariance of the Gaussian random vector (GRV) to at least second-order accuracy through the use of a set of sample points. UKF is an effective method to estimate the states or the parameters of a discrete dynamic system. In this paper, we use UKF to estimate VDPs through a two-stage method. The frame diagram of the two-stage estimation method is shown in Figure 3. In this paper, we assume that the velocity of the vehicle can be measured by GPS, and the vehicle mass is known. The driving or braking torques of vehicle ($T_{i}$) can be obtained. Longitudinal acceleration $a_{x}$, lateral acceleration $a_{y}$ and the yaw rate $\dot{\psi }$ can be measured by IMU. Rolling stiffness $k_{\phi }$ and roll damping $c_{\phi }$ are given by the manufacturer. The relevant parameters are listed in Table 1.

As shown in Figure 3, the longitudinal vehicle dynamics model was adopted during the first stage. For the first stage, the longitudinal vehicle dynamics model could be described by Equations (1)–(3), (11a) and (14). To make sure the longitudinal vehicle dynamic model is able to reflect the real state of the vehicle, the absolute value of the front-wheel steering angle needed to be smaller than **0.62 deg** and the absolute value of yaw rate needed to be smaller than **1 deg/s** (as shown in Table 1). The inputs of the longitudinal vehicle dynamics model were wheel torque $T_{i}\left(i=f,r\right)$. The states of the longitudinal vehicle dynamics model included the angular speed $\omega_{i}\left(i=f,r\right)$ and longitudinal vehicle velocity $v_{x}$. The measurable outputs were the angular speed $\omega_{i}\left(i=f,r\right)$ and longitudinal vehicle velocity $v_{x}$. As shown in Figure 3, the distance between the vehicle centroid and vehicle front $l_{f}$, the height of vehicle centroid h and the tire longitudinal stiffness $C_{K}$ are estimated parameters. To enable the VDPs to be estimated (persistent excitation requirement), specific command signals needed to be given to activate the corresponding parameters (As shown in Table 1).

When $l_{f}$, h and $C_{K}$ were estimated during the first stage, these estimated VDPs could be used in the second stage (as shown in Figure 3). During the second stage, a single-track with roll dynamics vehicle model was used and is described by Equations (4)–(12). As shown in Figure 3, the inputs of the single-track with the roll dynamics vehicle model the wheel steer angle δ, lateral accelerations $a_{y}$, yaw rate $\dot{\psi }$ and the roll rate $\dot{\phi }$. The states of this model include longitudinal vehicle velocity $v_{x}$, lateral velocity $v_{y}$, longitudinal accelerations $a_{x}$, lateral acceleration $a_{y}$, yaw rate $\dot{\psi }$ and the roll rate $\dot{\phi }$. The estimated parameters are the moment of inertia around the yaw axis $I_{z}$, the moment of inertia around the longitudinal axis $I_{x}$ and the tire cornering stiffness $C_{\alpha }$. When the conditions of the second stage in Table 2 are met, VDPs ($I_{z}$, $I_{x}$, $C_{\alpha }$) can be estimated.

As shown in Figure 3, UKF was used in both the first stage and second stage. UT is one of the most important parts of UKF. First, we introduce UT here. UT is shown in Table 2 [29].

When a system function is given as y=f(x), x is the state and the dimension of x is L (as shown in Table 2). Given an L-dimensional GRV x with mean $\hat{x}$ and covariance $P_{x}$, the statistics of y=f(x) were approximated by the selection of 2L+1 discrete sample points ${\left\{\chi_{i}\right\}}_{i=0}^{2L}=\left\{\hat{x}\;and\;\hat{x}\pm \sigma_{j},j=1,\ldots ,L\right\}$ where $\sigma_{j}$ is the $i^{th}$ column of the matrix $\sqrt{\left(L+\lambda \right)P_{x}}$. λ is a scaling parameter and depends on α, κ and L. The constant α determines the spread of sigma points about the mean $\hat{x}$. The constant κ is generally set to 3−L. The constant β was used to incorporate prior knowledge of the distribution. In this paper, α=0.01, β=2.

As shown in Table 3, ω represents VDPs; x represents the states of dynamics; d represents the measured vector; u represents the input vector of the dynamic system. $R_{k}^{e}$ is the measurement noise covariance. $R_{k}^{r}$ is the processing noise covariance. The corresponding parameters are shown in Table 4 and Section 4. At different stages, these variables represented different parameters. The local observability was demonstrated by investigating the rank of the observability matrix [20]. If the observability matrix had the full column rank, it was said to be locally observable. Using the continuous state-space representation, the discretized state-space representation was written by the Euler’s forward discretization in (15).

(15) $$d\left(x_{k}\right)=x_{k-1}+T_{s}G\left(x_{k-1},\omega_{k-1},u_{k-1}\right)$$

where $T_{s}$ is the sampling time. The observability matrix is the Jacobian of measurement vector d, with respect to the parameter vector ω. During the first stage, the Longitudinal vehicle dynamics model was used. According to Equations (1)–(3), (11a) and (14), the dynamic functions could be rewritten as:

(16) $$m{\dot{v}}_{x}=\left(mg\frac{l_{r}}{L}-ma_{x}\frac{h}{L}\right)C_{K}s_{f}+\left(mg\frac{l_{f}}{L}+ma_{x}\frac{h}{L}\right)C_{K}s_{r}$$

(17) $$J{\dot{\omega }}_{f}=T_{f}-r\left(mg\frac{l_{r}}{L}-ma_{x}\frac{h}{L}\right)C_{K}s_{f}$$

(18) $$J{\dot{\omega }}_{r}=T_{r}-r\left(mg\frac{l_{f}}{L}+ma_{x}\frac{h}{L}\right)C_{K}s_{r}$$

and the measurement vector ***d*** was written as:

(19) $$d=G_{1}(x,\omega_{1},u)=\left[\begin{array}{l} g_{11}\\ g_{12}\\ g_{13} \end{array}\right]=\left[\begin{array}{c} \left(g\frac{l_{r}}{L}-a_{x}\frac{h}{L}\right)C_{K}s_{f}+\left(g\frac{l_{f}}{L}+a_{x}\frac{h}{L}\right)C_{K}s_{r}\\ \frac{T_{f}-r(mg\frac{l_{r}}{L}-ma_{x}\frac{h}{L})C_{K}s_{f}}{J}\\ \frac{T_{r}-r(mg\frac{l_{f}}{L}+ma_{x}\frac{h}{L})C_{K}s_{r}}{J} \end{array}\right]$$

where $\omega_{1}$ represents a constant vector of the vehicle inertial parameters. During the first stage, $\omega_{1}={\left[\begin{array}{ccc} l_{f} & h & C_{k} \end{array}\right]}^{T}$. The observability matrix was defined as the Jacobian of $G_{1}$ with respect to the parameter vector $\omega_{1}$. The Jacobian matrix, $C_{1}=\nabla_{\omega_{1}}G_{1}$, was represented as:

(20) $$C_{1}=\nabla_{\omega_{1}}G_{1}=\left[\begin{array}{ccc} \frac{\partial g_{11}}{\partial \omega_{11}} & \frac{\partial g_{11}}{\partial \omega_{12}} & \frac{\partial g_{11}}{\partial \omega_{13}}\\ \frac{\partial g_{12}}{\partial \omega_{11}} & \frac{\partial g_{12}}{\partial \omega_{12}} & \frac{\partial g_{12}}{\partial \omega_{13}}\\ \frac{\partial g_{13}}{\partial \omega_{11}} & \frac{\partial g_{13}}{\partial \omega_{12}} & \frac{\partial g_{13}}{\partial \omega_{13}} \end{array}\right]$$

where

$$\frac{\partial g_{11}}{\partial \omega_{11}}=-\frac{g}{L}C_{K}s_{f}+\frac{g}{L}C_{K}s_{r},\frac{\partial g_{11}}{\partial \omega_{12}}=-\frac{a_{x}}{L}C_{K}s_{f}+\frac{a_{x}}{L}C_{K}s_{r},$$

$$\frac{\partial g_{11}}{\partial \omega_{13}}=gs_{f}-g\frac{l_{f}}{L}s_{f}-a_{x}\frac{h}{L}s_{f}+g\frac{l_{f}}{L}s_{r}+a_{x}\frac{h}{L}s_{r}$$

$$\frac{\partial g_{12}}{\partial \omega_{11}}=\frac{rmg}{JL}C_{K}s_{f},\frac{\partial g_{12}}{\partial \omega_{12}}=\frac{rma_{x}C_{K}s_{f}}{JL},\frac{\partial g_{12}}{\partial \omega_{13}}\;=-rmg\frac{1}{J}s_{f}+rmg\frac{l_{f}}{JL}s_{f}+ma_{x}r\frac{h}{JL}s_{f}$$

$$\frac{\partial g_{13}}{\partial \omega_{11}}=-\frac{rmgC_{K}s_{r}}{JL},\frac{\partial g_{13}}{\partial \omega_{12}}=-\frac{rma_{x}C_{K}s_{r}}{JL},\frac{\partial g_{13}}{\partial \omega_{13}}\;=-r(mg\frac{l_{r}}{JL}+ma_{x}\frac{h}{JL})s_{r}$$

Then $C_{1}$ could be written as:

(21) $$\begin{array}{l} C_{1}=\nabla_{\omega_{1}}G_{1}=\\ \left[\begin{array}{ccc} -\frac{g}{L}C_{K}s_{f}+\frac{g}{L}C_{K}s_{r} & -\frac{a_{x}}{L}C_{K}s_{f}+\frac{a_{x}}{L}C_{K}s_{r} & gs_{f}-g\frac{l_{f}}{L}s_{f}-a_{x}\frac{h}{L}s_{f}+g\frac{l_{f}}{L}s_{r}+a_{x}\frac{h}{L}s_{r}\\ \frac{rmgC_{K}s_{f}}{JL} & \frac{rma_{x}C_{K}s_{f}}{JL} & -rmg\frac{1}{J}s_{f}+rmg\frac{l_{f}}{JL}s_{f}+ma_{x}r\frac{h}{JL}s_{f}\\ -\frac{rmgC_{K}s_{r}}{JL} & -\frac{rma_{x}C_{K}s_{r}}{JL} & -r(mg\frac{l_{r}}{JL}+ma_{x}\frac{h}{JL})s_{r} \end{array}\right] \end{array}$$

As shown in the above equation, the observability matrix was able to meet the requirement of the full column rank as long as the acceleration $a_{x}$ was properly selected.

During the second stage, the measurement vector consisted of the longitudinal vehicle velocity, yaw rate and roll rate. According to Equations (4)–(14), the dynamic functions could be rewritten as:

(22) $${\dot{v}}_{x}=\frac{1}{m}\left(C_{\sigma }s_{f}cos\delta +C_{\alpha }\alpha_{f}sin\delta +C_{\sigma }s_{r}\right)+v_{y}\dot{\psi }$$

(23) $$\ddot{\psi }=\frac{l_{f}\left(C_{\sigma }s_{f}cos\delta +C_{\alpha }\alpha_{f}sin\delta \right)-l_{r}C_{\alpha }\alpha_{r}}{I_{z}}$$

(24) $$\ddot{\phi }=\frac{h\left(C_{\sigma }s_{f}sin\delta +C_{\alpha }\alpha_{f}cos\delta +C_{\alpha }\alpha_{r}\right)+mgh\phi -k_{\phi }\phi -c_{\phi }\dot{\phi }}{I_{x}}$$

then the measurement vector d was written as:

(25) $$\begin{array}{l} d=G_{2}\left(x,\omega_{2}\right)=\left[\begin{array}{c} g_{21}\\ g_{22}\\ g_{23} \end{array}\right]\\ =\left[\begin{array}{c} \frac{1}{m}\left(C_{\sigma }s_{f}cos\delta +C_{\alpha }\alpha_{f}sin\delta +C_{\sigma }s_{f}\right)+v_{y}\dot{\psi }\\ \frac{l_{f}\left(C_{\sigma }s_{f}cos\delta +C_{\alpha }\alpha_{f}sin\delta \right)-l_{r}C_{\alpha }\alpha_{r}}{I_{z}}\\ \frac{h\left(C_{\sigma }s_{f}sin\delta +C_{\alpha }\alpha_{f}cos\delta +C_{\alpha }\alpha_{r}\right)+mgh\phi -k_{\phi }\phi -c_{\phi }\dot{\phi }}{I_{x}} \end{array}\right] \end{array}$$

where $\omega_{2}$ represents a constant vector of the vehicle inertial parameters. During the second stage, $\omega_{2}={\left[\begin{array}{ccc} I_{z} & I_{x} & C_{\alpha } \end{array}\right]}^{T}$. The observability matrix was defined as the Jacobian of $G_{2}$ with respect to the parameter vector $\omega_{2}$. The Jacobian matrix, $C_{2}=\nabla_{\omega_{2}}G_{2}$, was represented as:

(26) $$C_{2}=\nabla_{\omega_{2}}G_{2}=\left[\begin{array}{ccc} \frac{\partial g_{21}}{\partial \omega_{21}} & \frac{\partial g_{21}}{\partial \omega_{22}} & \frac{\partial g_{21}}{\partial \omega_{23}}\\ \frac{\partial g_{22}}{\partial \omega_{21}} & \frac{\partial g_{22}}{\partial \omega_{22}} & \frac{\partial g_{22}}{\partial \omega_{23}}\\ \frac{\partial g_{23}}{\partial \omega_{21}} & \frac{\partial g_{23}}{\partial \omega_{22}} & \frac{\partial g_{23}}{\partial \omega_{23}} \end{array}\right]$$

where

$$\frac{\partial g_{21}}{\partial I_{z}}=0,\frac{\partial g_{21}}{\partial I_{x}}=0,\frac{\partial g_{21}}{\partial C_{\alpha }}=\frac{\alpha_{f}sin\delta }{m}$$

$$\frac{\partial g_{22}}{\partial I_{z}}=-\frac{l_{f}\left(C_{\sigma }s_{f}cos\delta +C_{\alpha }\alpha_{f}sin\delta \right)-l_{r}C_{\alpha }\alpha_{r}}{I_{z}^{2}},\frac{\partial g_{22}}{\partial I_{x}}=0,\frac{\partial g_{22}}{\partial C_{\alpha }}=\frac{l_{f}\alpha_{f}sin\delta -l_{r}\alpha_{r}}{I_{z}}.$$

$$\frac{\partial g_{23}}{\partial I_{z}}=0,\frac{\partial g_{23}}{\partial I_{x}}=-\frac{h\left(C_{\sigma }s_{f}sin\delta +C_{\alpha }\alpha_{f}cos\delta +C_{\alpha }\alpha_{r}\right)+mhg\phi -k_{\phi }\phi -c_{\phi }\dot{\phi }}{I_{x}^{2}},\frac{\partial g_{23}}{\partial C_{\alpha }}\;=\frac{h\alpha_{f}cos\delta +h\alpha_{r}}{I_{x}}$$

Then $C_{2}$ could be written as:

(27) $$\begin{array}{l} C_{2}=\nabla_{\omega_{2}}G_{2}\\ =\left[\begin{array}{ccc} 0 & 0 & \frac{\alpha_{f}sin\delta }{m}\\ -\frac{l_{f}\left(C_{\sigma }s_{f}cos\delta +C_{\alpha }\alpha_{f}sin\delta \right)-l_{r}C_{\alpha }\alpha_{r}}{I_{z}^{2}} & 0 & \frac{l_{f}\alpha_{f}sin\delta -l_{r}\alpha_{r}}{I_{z}}\\ 0 & -\frac{h\left(C_{\sigma }s_{f}sin\delta +C_{\alpha }\alpha_{f}cos\delta +C_{\alpha }\alpha_{r}\right)+mhg\phi -k_{\phi }\phi -c_{\phi }\dot{\phi }}{I_{x}^{2}} & \frac{h\alpha_{f}cos\delta +h\alpha_{r}}{I_{x}} \end{array}\right] \end{array}$$

As shown in the above equation, the observability matrix was able to meet the requirement of full column rank as long as the steering angle δ was properly selected. Based on the above analysis, the VDPs can be estimated when the acceleration $a_{x}$ and steering angle δ are designed according to Table 2.

In order to compare the estimation performance of the method proposed in this paper, we used the commonly used extended Kalman algorithm for comparison. The extended Kalman algorithm is shown in Table 5. 

The meanings of relevant parameters in the Table 5 are same as the meanings of relevant parameters in Table 4.

## 4. Simulation Results

The parameters of the vehicle model are shown in Table 6.

**Figure 4:**
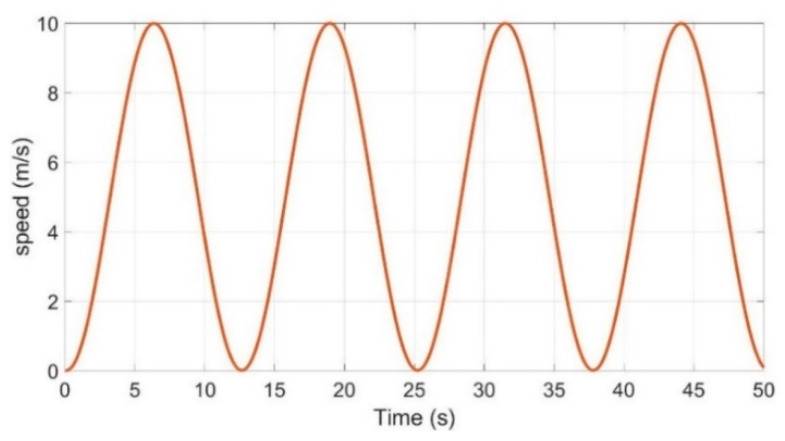
Vehicle longitudinal speed command.

As shown in Figure 3, the whole parameter estimation process was divided into two parts. The second stage of the estimation could only start after the first stage of the estimation was completed. Due to space limitations, the control of the vehicle is not discussed here. The vehicle control can refer to reference [36,37,38,39]. First, we estimated $I_{z},I_{x},C_{\alpha }$. As shown in Table 1, the vehicle needed to be continuously accelerated and braked for VDPs estimation. For this paper, the vehicle speed command signal was set as shown in Figure 4. It is a sinusoidal signal with a period of 12.5 s and an amplitude of 10. It includes acceleration/deceleration and can meet the requirements of the first stage (as shown in Table 1). 

**Figure 5:**
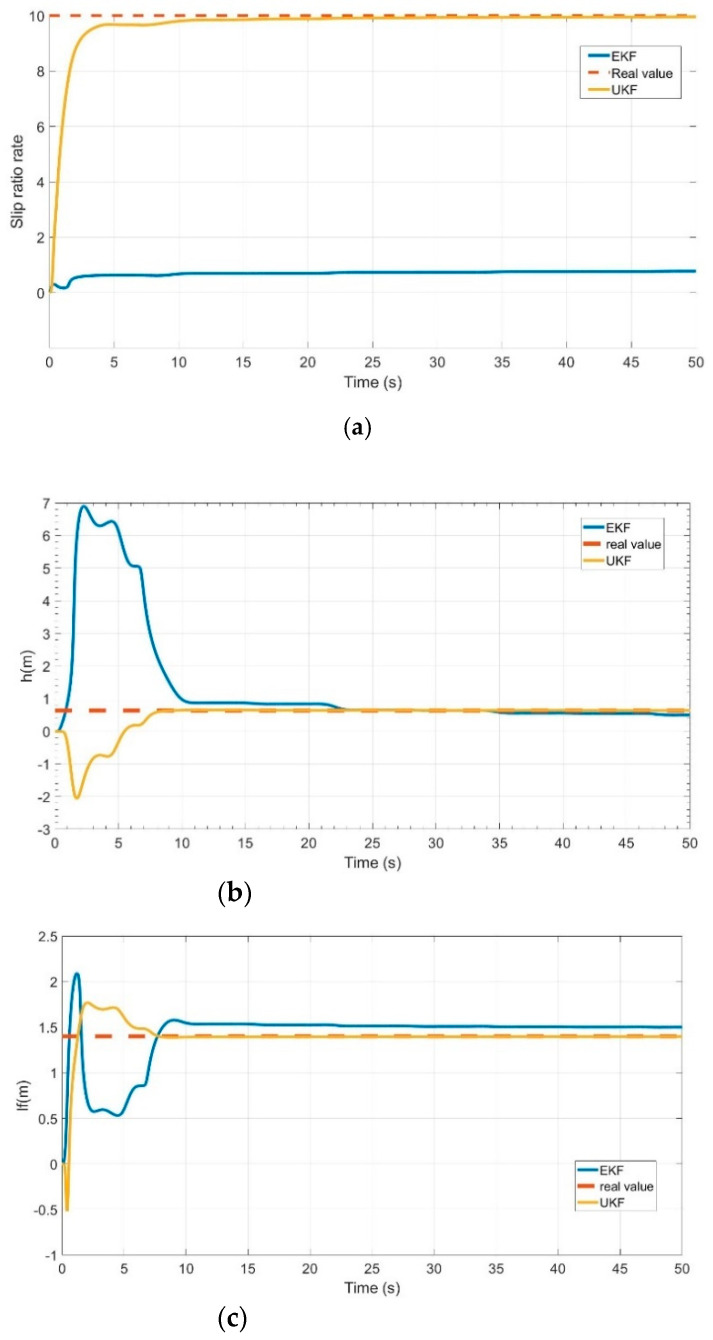
First stage VDPs estimation: (**a**) slip ratio rate estimation; (**b**) the height of vehicle centroid estimation; (**c**) estimation of the distance between the vehicle centroid and vehicle front axis.

VDPs can be estimated by making the car follow the command signal (as shown in Figure 4) to run for 4 cycles and 50 s. The simulation results are shown in Figure 5.

**Figure 6:**
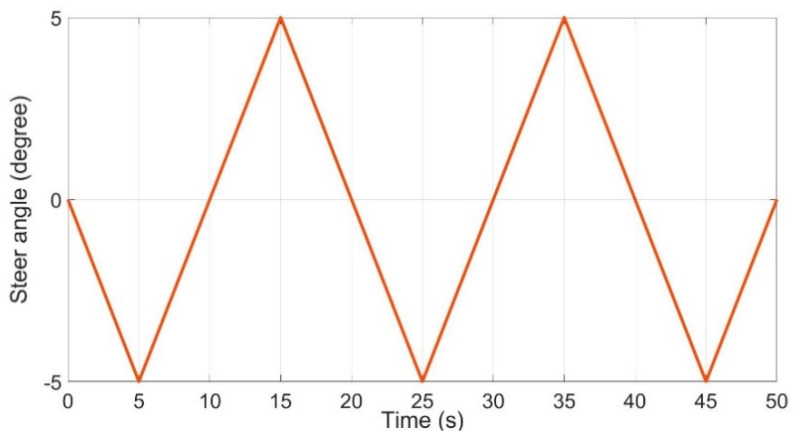
Vehicle steering angle command signal.

As shown in Figure 5, the slip ratio rate $C_{\sigma }$,the height of vehicle centroid *h* and the distance between the vehicle centroid and vehicle front axis $l_{f}$ were estimated and the estimated values approximated the real values in a short time (about 10 s) through the proposed method in this paper. Compared with the UKF used in this paper, the estimation error of EKF was larger (as shown in Figure 5). When $C_{\sigma }$, h and $l_{f}$ were estimated, they were used in the second stage. To meet the requirement of the second stage (as shown in Table 2), the vehicle steering angle command signal was set as Figure 6. 

**Figure 7:**
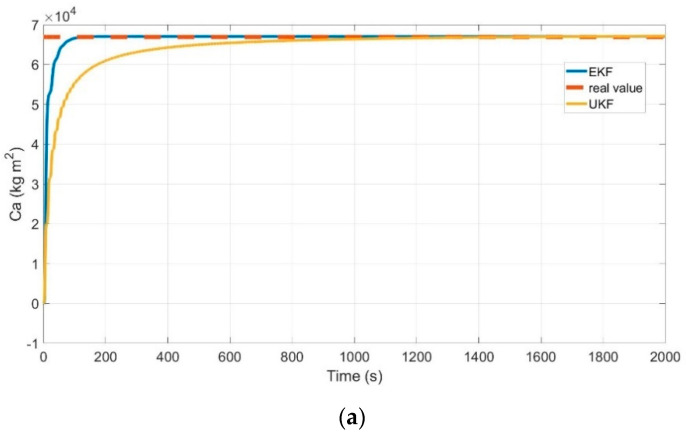

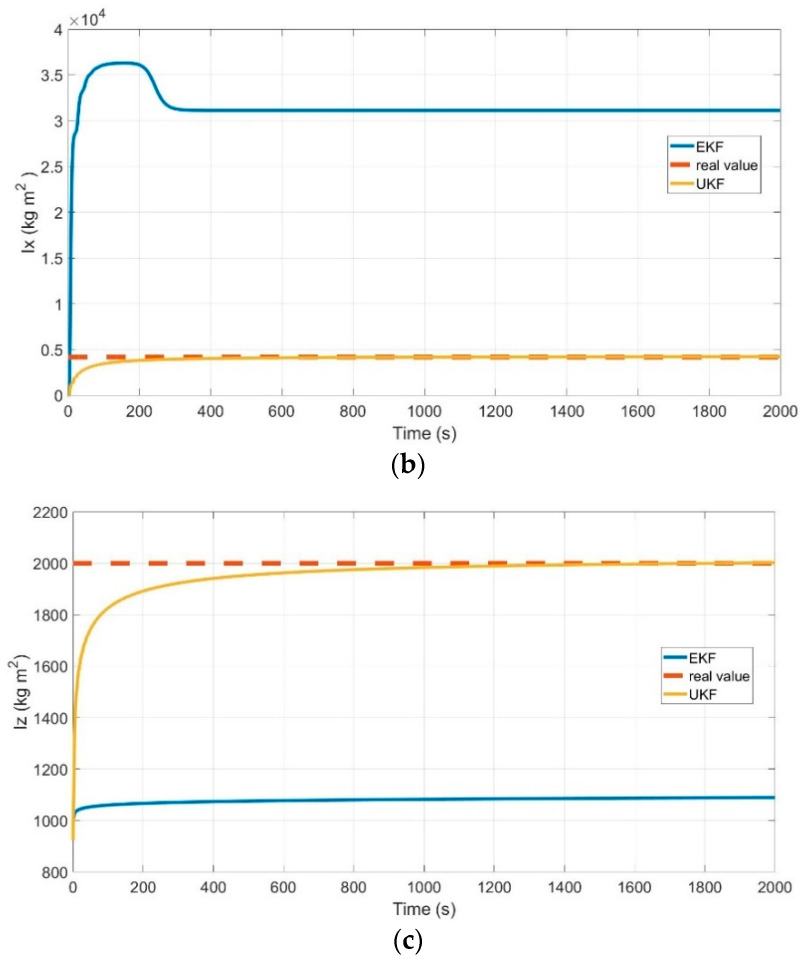
Second stage VDPs estimation. (**a**) the tire cornering stiffness estimation (**b**) the moment of inertia around the longitudinal axis estimation (**c**) the moment of inertia around the yaw axis estimation.

As shown in Figure 6, the vehicle steering angle command signal is a sawtooth wave with a period of 10 s and an amplitude of 5. Additionally, the vehicle operated at a speed of 10 m/s. The simulation results are shown in Figure 7.

As shown in Figure 7, tire cornering stiffness $C_{\alpha }$, the moment of inertia around the longitudinal axis $I_{x}$ and the moment of inertia around the yaw axis $I_{y}$ can be well estimated and the estimated error is small through the method proposed by us. However, the estimation error by EKF becomes larger compared with the first stage (as shown in Figure 7). The simulation results show that the proposed method is very capable of estimating the VDPs, and thus proves the effectiveness of the proposed method. This is mainly caused by two reasons. First, the estimated parameters with larger errors in the first stage are used in the second stage. Second, the parameter estimation in the second stage is a non-linear estimation (This can be seen in Equation (27)). The two simulation results prove that the method proposed in this paper can more accurately estimate the VDPs.

## 5. Discussion and Conclusions

In this paper, a new method is proposed to estimate VDPs. Different from other studies that only estimated portions of VDPs, the proposed two-stage estimation method which combines multiple-models and the Unscented Kalman Filter is able to estimate more VDPs. Because the states of a vehicle are affected by the tire stiffness, the tire stiffness is difficult to measure. The proposed estimation method is able to estimate VDPs and tire stiffness. The proposed two-stage estimation method also solves the problem that VDPs have a coupling effect on vehicle motion, which makes the VDPs difficult to estimate. For comparison, EKF is used. The simulation results prove that the proposed method not only can estimate VDPs but also that the estimation errors are small. 

The proposed two-stage estimation method in this paper can obtain all the VDPs needed for vehicle dynamics modeling at one time. It is useful for vehicle modeling, control and autonomous driving control algorithm tests on a test rig. More and more artificial intelligence technologies are being widely used in autonomous driving. However, most intelligent control algorithms are trained using vehicle kinematics models. An intelligent control algorithm trained with the kinematics model cannot accurately reflect the state of a real vehicle on the road. In order to ensure the effectiveness of the intelligent control algorithm, the vehicle dynamics model needs to be used in the algorithm training process. However, VDPs provided by most vehicle and devices manufacturers are not complete. The method proposed in this paper can estimate most of the VDPs required for vehicle dynamics modeling. Then it can be used to develop intelligent control algorithms for autonomous vehicles.

Our current research work verifies the effectiveness of the method proposed in this paper from the simulation. It verifies the program in advance for the next step of real vehicle test verification. In addition, it is assumed that some vehicle states can be measured directly in this paper. However, they are difficult to obtain in real scenarios. In the future work, we will use a Dual Unscented Kalman Filter to estimate the unmeasurable states and VDPs simultaneously.

## Figures and Tables

**Table 1:** Nomenclature.

Parameter	Description
*m*	Vehicle mass
*g*	Gravitational constant
*I_x_*	The moment of inertia around the longitudinal axis
*I_z_*	The moment of inertia around the yaw axis
*b*	Vehicle width
*l_f_*	Distance between the vehicle centroid and vehicle front axis
*l_r_*	Distance between the vehicle centroid and vehicle rear axis
*r*	Effective tire radius
*h*	Height of vehicle centroid
*c_ϕ_*	Roll damping coefficient
*k_ϕ_*	Roll stiffness
*C_α_*	Tire cornering stiffness
*C_K_*	Slip ratio rate
*J*	Wheel moment of inertia
*v_x_*	Longitudinal vehicle velocity
*F_xf_*	Longitudinal forces of the front tire
*F_xr_*	Longitudinal forces of the rear tire
*T_f_*	Front wheel torque
*T_r_*	Rear wheel torque
*F_zf_*	Vertical force of front wheel
*F_zr_*	Vertical force of rear wheel
*a_x_*	Longitudinal accelerations
$\dot{\psi }$	Yaw rate
$\dot{\phi }$	Roll rate
*a_y_*	Lateral acceleration
*δ*	Wheel steer angle
*F_yr_*	Lateral forces of the rear tires
*C_σ_*	Tire longitudinal stiffness
*S_i_*	Slip ratio
*α_i_*	Slip angle

**Table 2:** Two-stage estimation condition requirements.

First Stage: Linear Acceleration/Deceleration
Longitudinal vehicle dynamic model
|Front Wheel steering angel|<0.62 deg
|Yaw Rate|<1deg/s
Longitudinal acceleration/deceleration
Second stage: Continuous turn
Single-track with roll dynamics vehicle model
First stage estimation finished
Longitudinal speed remains constant
Continuous turn

**Table 3:** UT.

UT Setup
$\lambda =\alpha^{2}\left(L+\kappa \right)-L$
$W_{0}^{\left(m\right)}=\frac{\lambda }{L+\lambda }$
$W_{0}^{\left(c\right)}=\frac{\lambda }{L+\lambda }+1-\alpha^{2}+\beta$
$W_{i}^{\left(m\right)}=W_{i}^{\left(c\right)}=\frac{1}{2\left(L+\lambda \right)}\;,\;\;\;i=1,\ldots ,2L$
$\gamma =\sqrt{L+\lambda }$

**Table 4:** UKF for VDPs estimation.

1: Initialize ${\hat{\omega }}_{0}^{+}$, $P_{\omega 0}^{+}$
${\hat{\omega }}_{0}^{+}=E\left[\omega \left(0\right)\right]$
$P_{\omega 0}^{+}=E\left[\left(\omega \left(0\right)-{\hat{\omega }}_{0}^{+}\right)\left(\omega \left(0\right)-{\hat{\omega }}_{0}^{+}\right)\right]$
2: Prediction and sigma-point calculation: ${\hat{\omega }}_{k}^{-}={\hat{\omega }}_{k-1}^{+}$
$P_{\omega_{k}}^{-}=P_{\omega_{k-1}}^{+}+R_{\omega_{k-1}}^{r}$
$W_{k|k-1}=\left[\begin{array}{ccc} {\hat{\omega }}_{k}^{-} & {\hat{\omega }}_{k}^{-}+\gamma \sqrt{P_{\omega_{k}}^{-}} & {\hat{\omega }}_{k}^{-}-\gamma \sqrt{P_{\omega_{k}}^{-}} \end{array}\right]$
$D_{k|k-1}=G\left(x_{k},W_{k|k-1},u_{k}\right)$
${\hat{d}}_{k}^{-}=\overset{2L}{\underset{i=0}{\sum }}W_{i}^{\left(m\right)}D_{i,k|k-1}$
3: Update after the measurement of d(k)
$P_{d_{k}}^{-}=\overset{2L}{\underset{i=0}{\sum }}W_{i}^{\left(c\right)}(D_{i,k|k-1}-{\hat{d}}_{k}^{-}){\left(D_{i,k|k-1}-{\hat{d}}_{k}^{-}\right)}^{T}+R_{k}^{e}$
$P_{\omega_{k}d_{k}}^{-}=\overset{2L}{\underset{i=0}{\sum }}W_{i}^{\left(c\right)}(W_{i,k|k-1}-{\hat{\omega }}_{k}^{-}){\left(D_{i,k|k-1}-{\hat{d}}_{k}^{-}\right)}^{T}$
$K_{k}=P_{\omega_{k}d_{k}}^{-}{\left(P_{d_{k}}^{-}\right)}^{-1}$
${\hat{\omega }}_{k}^{+}={\hat{\omega }}_{k}^{-}+K_{k}\left[d\left(k\right)-{\hat{d}}_{k}^{-}\right]$
$P_{\omega_{k}}^{+}=P_{\omega_{k}}^{-}-K_{k}P_{d_{k}}^{-}K_{k}^{T}$

**Table 5:** EKF for VDPs estimation.

EKF Algorithm
1. Initialize ${\hat{\omega }}_{0}^{+}$, $P_{\omega_{0}}^{+}$
${\hat{\omega }}_{0}^{+}=E\left[\omega \left(0\right)\right]$
$P_{\omega_{0}}^{+}=E\left[\left(\omega \left(0\right)-{\hat{\omega }}_{0}^{+}\right)\left(\omega \left(0\right)-{\hat{\omega }}_{0}^{+}\right)\right]$
2. Prediction before the measurement of d(k)
${\hat{\omega }}_{k}^{-}={\hat{\omega }}_{k-1}^{+}$
$P_{\omega_{k}}^{-}=P_{\omega_{k-1}}^{+}+R_{k-1}^{r}$
${\hat{d}}_{k}^{-}=G\left({\hat{\omega }}_{k-1}^{+},s\left(k-1\right),u\left(k-1\right)\right)$
3. Update after the measurement of d(k)
${Κ}_{k}=P_{\omega_{k}}^{-}C_{k}^{-}{}^{T}{\left(C_{k}^{-}P_{\omega_{k}}^{-}C_{k}^{-}{}^{T}+R_{k}^{e}\right)}^{-1}$
${\hat{\omega }}_{k}^{+}={\hat{\omega }}_{k}^{-}+{Κ}_{k}\left[d_{k}-{\hat{d}}_{k}^{-}\right]$
$P_{\omega_{k}}^{+}=P_{\omega_{k}}^{-}-{Κ}_{k}C_{k}^{-}P_{\omega_{k}}^{-}$

**Table 6:** Model parameters and definitions.

Parameter	Description	Value	Unit
m	Vehicle mass	1600	kg
g	Gravitational constant	9.8	$m/s^{2}$
$I_{x}$	The moment of inertia around the longitudinal axis	4175	${\mathrm{kg}\;m}^{2}$
$I_{z}$	The moment of inertia around the yaw axis	2000	${\mathrm{kg}\;m}^{2}$
b	Vehicle width	1.53	m
$l_{f}$	Distance between the vehicle centroid and vehicle front axis	1.4	m
$l_{r}$	Distance between the vehicle centroid and vehicle rear axis	1.1	m
r	Effective tire radius	0.3	m
h	Height of vehicle centroid	0.637	m
$c_{\phi }$	Roll damping coefficient	5737	Nms/deg
$k_{\phi }$	Roll stiffness	36,000	Nm/deg
$C_{\alpha }$	Tire cornering stiffness	66,900	N/rad
$C_{K}$	Slip ratio rate	10	-
J	Wheel moment of inertia	0.6	${\mathrm{kg}\;m}^{2}$

## Data Availability

Not applicable.
